# Hemodynamic management of acute brain injury caused by cerebrovascular diseases: a survey of the European Society of Intensive Care Medicine

**DOI:** 10.1186/s40635-022-00463-6

**Published:** 2022-09-12

**Authors:** Antonio Messina, Federico Villa, Giulia Lionetti, Laura Galarza, Geert Meyfroidt, Mathieu van der Jagt, Xavier Monnet, Paolo Pelosi, Maurizio Cecconi, Chiara Robba

**Affiliations:** 1 Department of Anesthesia and Intensive Care Medicine, IRCCS Humanitas Research Hospital – IRCCS, Humanitas University, via Alessandro Manzoni 56, 20089 Rozzano, Milan Italy; 2 Department of Biomedical Sciences, Humanitas University, Pieve Emanuele, Milan Italy; 3 Department of Intensive Care, Hospital General Universitario de Castellon, Castellon de la Plana, Spain; 4 Department and Laboratory of Intensive Care Medicine, University Hospitals Leuven and KU Leuven, Louvain, Belgium; 5 Department of Intensive Care Adults and Erasmus MC Stroke Center, Erasmus MC – University Medical Center, Rotterdam, The Netherlands; 6 Paris-Saclay University, AP-HP, Medical Intensive Care Unit, Bicêtre Hospital, DMU CORREVE, Inserm UMR S_999, FHU SEPSIS, CARMAS Research Team, Le Kremlin-Bicêtre, France; 7 Anaesthesia and Intensive Care, San Martino Policlinico Hospital, IRCCS for Oncology and Neuroscience, Genoa, Italy; 8 Department of Surgical Sciences and Integrated Sciences, University of Genoa, Genoa, Italy

**Keywords:** Subarachnoid hemorrhage, Acute ischemic stroke, Intracranial hemorrhage, Hemodynamic management

## Abstract

**Background:**

The optimal hemodynamic targets and management of patients with acute brain injury are not completely elucidated, but recent evidence points to important impact on clinical outcomes. We performed an international survey with the aim to investigate the practice in the hemodynamic targets, monitoring, and management of patients with acute ischemic stroke (AIS), intracranial hemorrhage (ICH) and subarachnoid hemorrhage (SAH).

**Methods:**

This survey was endorsed by the European Society of Intensive Care (ESICM). An electronic questionnaire of 76 questions divided in 4 sections (general information, AIS, ICH, SAH specific questions) was available between January 2022 to March 2022 on the ESICM website.

**Results:**

One hundred fifty-four healthcare professionals from 36 different countries and at least 98 different institutions answered the survey. Routine echocardiography is routinely performed in 37% of responders in AIS, 34% in ICH and 38% in SAH. Cardiac output monitoring is used in less than 20% of cases by most of the responders. Cardiovascular complications are the main reason for using advanced hemodynamic monitoring, and norepinephrine is the most common drug used to increase arterial blood pressure. Most responders target fluid balance to neutral (62% in AIS, 59% in ICH,44% in SAH), and normal saline is the most common fluid used. Large variability was observed regarding the blood pressure targets.

**Conclusions:**

Hemodynamic management and treatment in patients with acute brain injury from cerebrovascular diseases vary largely in clinical practice. Further research is required to provide clear guidelines to physicians for the hemodynamic optimization of this group of patients.

## Take-home messages

- Heterogeneity exists in the approach of hemodynamic management of cerebrovascular disease.
- Pharmacologic strategies to achieve targets and monitoring targets are different across centers.
- Advanced, serial hemodynamic monitoring is not a standard of care in brain injured patients and it is reserved to most severe cases, although benefit might extend to hemodynamically stable patients.

## Introduction

The hemodynamic management of cerebrovascular diseases is of fundamental importance in order to minimize secondary brain damage [1, 2]. Cerebral autoregulation may be impaired in acute brain injured (ABI) patients such as ischemic stroke (AIS), intracranial hemorrhage (ICH) and subarachnoid hemorrhage (SAH), and consequently brain perfusion and its oxygenation might be compromised. In these pathologies, cerebral blood flow is extremely sensitive to systemic blood variation, and therefore suboptimal hemodynamic management (including fluids and vasoactive administration policy and hemodynamic monitoring) of these patients may increase ischemic areas, promote hemorrhagic evolution and develop secondary brain injury [2–4].

Clinical strategies of hemodynamic management of these pathologies are still based on moderate/low quality of evidence retrieved from relatively small studies, and no strong recommendations are reported in the most updated consensuses on these topics [5–9]. Both the hemodynamic monitoring/targets and fluid management/balance should consider the physiological peculiarities of ABI patients, in particular given the disturbed cerebral autoregulation, tendency for increased intracranial pressure (ICP) and decreased cerebral perfusion pressure (CPP) and in those affected by an acute cardiac dysfunction associated with sympathetic stimulation and catecholamine release [9].

In the AIS the choice of the optimal pressure target is still debated. A recent multicentric randomized trial did not find difference in long-term clinical outcomes in AIS patients between intensive [target systolic arterial pressure (SAP) 130–140 mm Hg within 1 h] or “standard” (SAP < 180 mm Hg) blood pressure-lowering treatment over 72 h [10]. In ICH patients, despite contrasting results of trials investigating hemodynamic management, monitoring, as well as the targets of blood pressure to be achieved, the most recent guidelines recommend lowering SAP to a target range of 130 to 140 mm Hg in patients presenting with acute ICH of mild to moderate severity and SBP between 150 and 220 mm Hg [11–13]. In SAH patients, historical concepts of hemodynamic management, such as the “triple H” approach (hypertension, hypervolemia, and hemodilution) [14] have been challenged [15, 16]. The aim of this survey is to investigate the current hemodynamic practices worldwide for the management of AIS, ICH and SAH.

## Methods

This is an international survey, proposed by the Neuro-intensive Care (NIC) and the Cardiovascular Dynamics (CD) sections of the European Society of Intensive Care Medicine (ESICM). No ethical approval was necessary for the development of this survey. The Steering committee, which included representatives of both ESICM sections, performed a non-systematic review of the literature (i.e., guidelines and consensus papers) on hemodynamic management of AIS, ICH and SAH patients and created a questionnaire of 76 questions divided in 4 sections (general information, acute ischemic stroke, intracranial hemorrhage, subarachnoid hemorrhage). The survey was reviewed and approved by the Research committee of the ESICM and was tested in a pilot cohort of potential respondents, including the Steering Committee. The survey was available on the ESICM website between January 2022 to March 2022. Three reminders were sent to potential responders. The questionnaire was created considering some issues around this topic, such as low levels of evidence, lack of good-quality studies and controversial results from observational trials. The survey was designed to identify (a) characteristics of the participants demographics, type of hospital/specialty and available neuromonitoring tools; (b) protocols for the hemodynamic management of this population; (c) hemodynamic targets used in this group of patients; (d) clinical management and use of hemodynamic monitoring in this population. Advanced hemodynamic monitoring was defined as the use of any further tool able to estimate cardiac output (i.e., calibrated/uncalibrated hemodynamic monitoring based on arterial waveform analysis; bioreactance technology, etc.).

The target audience was ESICM members who had agreed to participate in ESICM surveys at the time of their membership registration and who treat patients with cerebrovascular diseases in their clinical practice. The investigators invited NIC or CD sections members, asking them to involve more respondents locally. Participants did not receive compensation for their participation in the survey, which was distributed via the ESICM office, thus protecting data confidentiality and anonymity. The survey was registered within the ESICM Survey portfolio.

### Statistical methods

Data from the questionnaire were exported as a comma-separated value report from the Surveymonkey® software package and subsequently stored as an Excel file (Microsoft Corp, Redmond, WA). Descriptive statistics were computed for all study variables. The results are presented as numbers and percentage. Continuous variables are reported as mean [standard deviation (SD)] or median [interquartile range (IQR)], whereas categorical variables were reported as frequency and proportion.

## Results

### General characteristics

As shown in Table 1, 154 healthcare professionals from 36 different countries [87.1% from Europe] answered this survey. Answers came from 98 different institutions [mainly academic/teaching hospitals (83.1%) and neurocritical care centers (78.6%)] with different capacity, [most of them with > 1,000 patients hospital capacity (28.0%)]. Most of healthcare professionals who responded generally manage > 30 AIS (45.5%), ICH (46.1%) and SAH (55.2%) patients per year.

**Table 1:** General characteristics of participants

General information						
World region of responders						
**Europe**	**Australia**	**South America**	**India**	**Indonesia**	**Arabic countries**	**North America**
128 (87.1%)	1 (0,7%)	5 (3,4%)	7 (4,8%)	1 (0,7%)	2 (1,4%)	3 (2%)
**Type of institution:**						
**Academic/teaching hospital**	**District Hospital**	**Non-teaching hospital**	**Private non-academic hospital**			
128 (83,1%)	3 (1,9%)	19 (12,3%)	4 (2,6%)			
**Number of beds in your institution:**						
** < 250**	**250–500**	**500–750**	**750–1000**	** > 1000**		
14 (9,1%)	27 (17,5%)	33 (21,4%)	37 (24%)	43 (28%)		
**Catchment area population:**						
** < 100,000**	**100,000–250,000**	**250,000–500,000**	**500,000–750,000**	**750,000–1,000,000**	** > 1,000,000**	
9 (5,8%)	24 (15,6%)	29 (18,8%)	30 (19,5%)	18 (11,7%)	44 (28,6%)	
**Are you a center for neurocritical care?**						
**Yes**	**No**					
121 (78,6%)	33 (21,4%)					
**Critically ill neuroscience patients are generally admitted to:**						
**Medical ICU**	**Mixed general-neurocritical care unit**	**Specialist neurocritical care unit**	**Surgical ICU**	**Other**		
14 (9,1%)	82 (53,2%)	45 (29,2%)	8 (5,2%)	5 (3,2%)		
**Number of acute ischemic stroke patients admitted to ICU /year:**						
**Less than 5**	**5 to 10**	**10 to 20**	**20–30**	** > 30**		
3 (1,9%)	20 (13%)	39 (25,3%)	22 (14,3%)	70 (45,5%)		
**Number of acute hemorrhagic stroke patients admitted to ICU /year:**						
**Less than 5**	**5 to 10**	**10 to 20**	**20–30**	** > 30**		
5 (3,2%)	17 (11%)	30 (19,5%)	31 (20,1%)	71 (46,1%)		
**Number of subarachnoid hemorrhage patients admitted to ICU /year:**						
**Less than 5**	**5 to 10**	**10 to 20**	**20–30**	** > 30**		
8 (5,2%)	20 (13%)	27 (17,5%)	14 (9,1%)	85 (55,2%)		
**Medical staffing of Neurocritical care unit/ICU admitting neurocritical care patients:**						
**Anesthetist intensivist**	**General/respiratory medicine Intensivist**	**Neurologist Intensivist**	**Neurosurgeon Intensivist**	**Other**		
86 (55,8%)	35 (22,7%)	17 (11%)	2 (1,3%)	14 (9,1%)		
**Medical staff present 24/7:**						
**Qualified specialist**	**Fellow**	**Trainee**	**Specialist nurse**	**Telepresence**	**Other**	**None**
134 (39%)	69 (20%)	68 (20%)	64 (19%)	5 (2%)	6 (2%)	0

ICU, intensive care unit

According to the responders, most of the patients with neurological critical illness are admitted to mixed general-neurocritical care unit (53%), and less commonly to specialist neurocritical care unit (29%) or other ICUs/wards. The majority of neurocritical patients are managed by anesthesiology intensivist (56%), followed by general/respiratory medicine intensivist (23%) and neurology intensivist (11%). Finally, in most of the centers (39%) a qualified specialist is 24/7 present.

### Hemodynamic management in AIS patients

A standardized protocol for arterial blood pressure management in patients with AIS is used by the 46% of the responders.

The use of echocardiography is quite heterogeneous: most of the participants (37%) declared to perform routine echocardiography during the first 24 h to rule out possible acute left ventricular dysfunction, while 9% only in selected cases. Cardiac output monitoring is used in less than 20% of cases by most (62%) of the participants; capillary refill time is used by less than half of the participants (40%) (Fig. 2).

An arterial pressure target of < 160/90 mmHg is reported in the 35% of patients eligible for thrombolysis, and in the 30% of patients after mechanical thrombectomy. In the population not eligible for intravenous thrombolysis, blood pressure targets of < 160/90 mmHg were reported in 29% of responders and < 185/110 mmHg in 27% of responders.

**Fig. 1:**
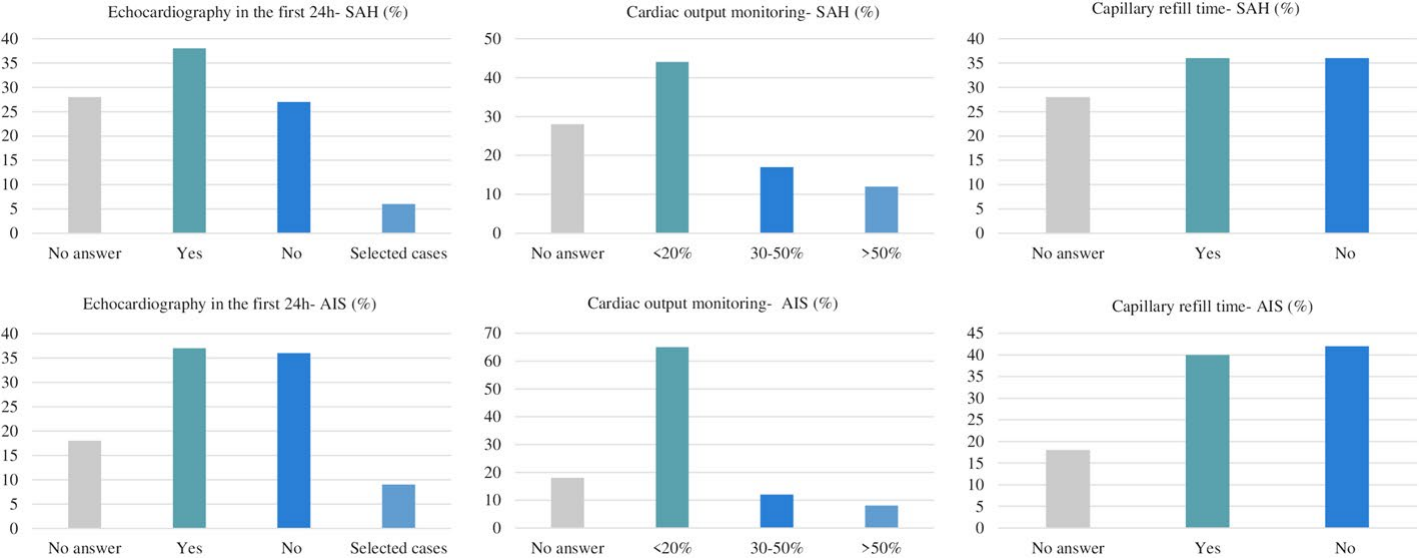
Hemodynamic monitoring in the considered subgroup of patients. Data are expressed as percentage of the overall answers obtained by the survey

In AIS patients admitted to ICU, hemodynamic monitoring is mostly performed (60%) by electrocardiogram (ECG) and invasive blood pressure (IBP), while less frequently by non-invasive blood pressure, NIBP and ECG (34%). Advanced hemodynamic monitoring is rarely used (10%) (Table 2 and Fig. 1).

**Table 2:** Questions and answers on acute ischemic stroke

Which is the percentage of acute ischemic stroke (AIS) that undergo pharmacological thrombolysis/year in your institution?								
**No answer**	** < 10%**	**10–30%**	**30–50%**	** > 50%**				
28 (18%)	23 (15%)	37 (24%)	28 (18%)	38 (25%)				
**Which is the percentage of AIS that undergo mechanic thrombectomy/year in your institution?**								
**No answer**	** < 10%**	**10–30%**	**30–50%**	** > 50%**				
28 (18%)	48 (31%)	32 (21%)	29 (19%)	17 (11%)				
**Do you have a standardized protocol for arterial blood pressure management in patients with ischemic stroke?**								
**No answer**	**Yes**	**No**						
28 (18%)	71 (46%)	55 (36%)						
**Do you routinely perform an echocardiography at the bedside in AIS patients during the first 24 h of admission to rule out possible acute LV dysfunction?**								
**No answer**	**Yes**	**No**	**Selected cases**					
28 (18%)	57 (37%)	55 (36%)	14 (9%)					
**How many AIS patients receive standard cardiac output monitoring in your institution?**								
**No answer**	** < 20%**	**30–50%**	**50%**					
28 (18%)	95 (62%)	19 (12%)	12 (8%)					
**Do you usually assess capillary refill test at the bedside in AIS patients?**								
**No answer**	**Yes**	**No**						
28 (18%)	62 (40%)	64 (42%)						
**Which is the arterial blood pressure target that you use in patients eligible for iv thrombolysis?**								
**No answer**	** < 140/90**	** < 160/90**	**185/110**	**Reduction of 15% of baseline ABP**		**Other**		
28 (18%)	16 (10%)	54 (35%)	33 (21%)	15 (10%)		8 (5%)		
**Which is the arterial blood pressure target that you use in patients not eligible for iv thrombolysis?**								
**No answer**	** < 140/90**	** < 160/90**	**185/110**	**Reduction of 15% of baseline ABP**		**Other**		
28 (18%)	13 (8%)	44 (29%)	42 (27%)	17 (11%)		10 (7%)		
**Which is the arterial blood pressure target that you use in patients eligible after mechanical thrombectomy?**								
**No answer**	** < 140/90**	** < 160/90**	**185/110**	**Reduction of 15% of baseline ABP**		**Other**		
28 (18%)	28 (18%)	46 (30%)	26 (17%)	11 (7%)		15 (10%)		
**Which standard hemodynamic monitoring do you use in case of patient with ischemic stroke? (more than one anwer)**								
**No answer**	**Basic monitoring (NIBP, ECG)**	**Basic monitoring** **(invasive blood pressure, ECG)**		**Advanced non-invasive hemodynamic monitoring**		**Advanced invasive monitoring**	**Echocardiography**	
28 (18%)	53 (34%)	92 (60%)		16 (10%)		9 (6%)	44 (29%)	
**Which is the commonest indication for starting an advanced hemodynamic monitoring in acute ischemic stroke patients in your unit? (i.e., the use of more than invasive arterial blood pressure)?**								
**No answer**	**Patients developing cardiovascular complications (i.e., pulmonary edema, cardiogenic shock, etc.)**		**Sepsis/septic shock**	**Difficult blood pressure management**		**Patients developing neurological complications (i.e., neuroworsening)**		
28 (18%)	52 (34%)		39 (25%)	25 (16%)		10 (6%)		
**How do you initially manage hypotension in AIS patients?**								
**No answer**	**Mean arterial pressure-based protocol**	**No specific protocol**	**Fluids**	**Cardiac output-based protocol**				
28 (18%)	85 (55%)	21 (14%)	14 (9%)	6 (4%)				
**Which drugs are used as first line to increase blood pressure in your ICU in patients with preserved heart function?**								
**No answer**	**Norepinephrine**	**Dobutamine**	**Vasopressin**	**Metaraminol**				
28 (18%)	123 (80%)	2 (1%)	1 (< 1%)	1 (< 1%)				
**Which drugs are used as second line to increase blood pressure in your ICU in patients with preserved heart function?**								
**No answer**	**Vasopressin**	**Epinephrine**	**Dobutamine**	**Dopamine**	**Norepinephrine**	**Terlipressin**	**Phenylephrine**	**None of the above**
28 (18%)	58 (38%)	27 (18%)	19 (12%)	4 (3%)	5 (3%)	2 (1%)	1 (< 1%)	2 (1%)
**Which drugs are used as first line to decrease blood pressure in your ICU?**								
**No answer**	**Urapidil**	**Beta blockers**	**Calcium channel blockers**	**Clonidine**	**Nitrates**	**ACE inhibitors**	**Angiotensin receptor antagonists**	**Azamethonium**
28 (18%)	37 (24%)	33 (21%)	23 (15%)	21 (14%)	6 (4%)	3 (2%)	2 (1%)	1 (< 1%)
**Which drugs are used as second line to decrease blood pressure in your ICU?**								
**No answer**	**Beta blockers**	**Calcium channel blockers**	**Clonidine**	**Urapidil**	**Nitrates**	**ACE inhibitors**	**Angiotensin receptor antagonists**	**Hydralazine**
28 (18%)	34 (22%)	32 (21%)	27 (18%)	11 (7%)	10 (6%)	7 (5%)	4 (3%)	1 (< 1%)
**How frequently do you monitor fluid balance in patients with ischemic stroke?**								
**No answer**	**Every hour**	**Every two hours**	**Every 4 h**	**Every 8 h**	**Every 12 h**	**Every 24 h**	**We don’t monitor fluid balance**	
28 (18%)	48 (31%)	22 (14%)	9 (6%)	1 (1%)	22 (14%)	22 (14%)	2 (1%)	
**Which is your net daily fluid balance target (i.e., all fluids INPUT minus all fluids OUTPUT)?**								
**No answer**	**Negative**	**Neutral**	**Positive**					
28 (18%)	10 (6%)	96 (62%)	20 (13%)					
**Which is the first line fluid therapy you usually use?**								
**No answer**	**Ringer lactate**	**Normal saline**	**Plasmalyte**	**Albumin**	**Hypertonic saline**	**Other**		
28 (18%)	54 (35%)	49 (32%)	6 (4%)	3 (2%)	2 (1%)	30 (19%)		

ICU, intensive care unit; AIS, acute ischemic stroke; ABP, arterial blood pressure; NIBP, non-invasive blood pressure; ECG, electrocardiogram; ACE, angiotensin-converting enzyme

The most common indication for advanced hemodynamic monitoring was developing cardiovascular complications (34%), followed by sepsis/septic shock (25%), difficult blood pressure management (16%) and worsening of neurological symptoms (6%).

Initial management of low blood pressure is mostly based on MAP-based protocol (55%), no specific protocol is reported in the 14% of the answers, and norepinephrine is largely the most used first-line agent in the treatment of hypotension in AIS patients with preserved heart function (80%). The preferred first-line agent for lowering blood pressure is urapidil (24% of the answers), followed by beta-blockers (21%), calcium channel blockers (15%) and clonidine (14%).

Most of the participants monitor fluid balance every hour (31%) and a neutral fluid balance is the target according to 62% of the participants. A positive (13%) or negative (6%) balance are rarely defined as a hemodynamic target. When fluids administration is required, crystalloids [ringer lactate (35%) or normal saline (32%)] are usually preferred.

### Hemodynamic management in patients with hemorrhagic stroke

In ICH patients, a standardized protocol for arterial blood pressure management is used by the 43% of the responders. Echocardiography was reported to be performed by the 34% of the responders and CO monitoring is used in less than 20% of patients by the 53% of the responders. CRT is performed by 37% the participants. The commonest indication for advanced monitoring is the development of cardiovascular complications (35%), followed by the requirevent of vasoactive drugs (26%).

The most commonly reported arterial pressure target was < 140/90 mmHg (36%), followed by < 160/90 mmHg (29%). Hemodynamic monitoring is performed by NIBP and ECG in 12%, and IBP and ECG in the 52% of the answers. Advanced hemodynamic monitoring is rarely used (6% of responders).

Initial management of low blood pressure is usually performed by a MAP-based protocol (53%) and the first-line agent in the treatment of hypotension is norepinephrine (74%). Drugs used in these patients as first line for lowering arterial blood pressure are usually beta-blockers (21%), calcium channel blockers (15%) and clonidine (10%). Fluid balance is mostly monitored hourly (31%) and the majority (59%) of participants aim at a neutral daily fluid balance, with the preferred fluids being ringer lactate (33%) and normal saline (28%) (Table 3 and Fig. 1).

**Table 3:** Questions and answers on hemorrhagic stroke

Do you have a standardized protocol for arterial blood pressure management in patients with hemorrhagic stroke?								
**No answer**	**Yes**	**No**						
35 (23%)	66 (43%)	53 (34%)						
**Do you routinely perform an echocardiography at the bedside in patients with hemorrhagic stroke during the first 24 h of admission to rule out possible acute LV dysfunction?**								
**No answer**	**Yes**	**No**	**Selected cases**					
35 (23%)	52 (34%)	59 (38%)	8 (5%)					
**How many patients with hemorrhagic stroke receive cardiac output monitoring in your institution?**								
**No answer**	** < 20%**	**30–50%**	**50%**					
35 (23%)	82 (53%)	23 (15%)	9 (8%)					
**Do you usually assess CRT (capillary refill time) at the bedside in patients with hemorrhagic stroke as part of routinely hemodynamic assessment?**								
**No answer**	**Yes**	**No**						
35 (23%)	57 (37%)	62 (40%)						
**Which is the arterial blood pressure target that you use in patients after intracranial hemorrhage?**								
**No answer**	** < 140/90**	** < 160/90**	**185/110**	**Reduction of 15% of the initial arterial blood pressure**		**other**		
35 (23%)	55 (36%)	44 (29%)	7 (5%)	8 (5%)		5 (3%)		
**Which hemodynamic monitoring do you use in case of patient with hemorrhagic stroke?**								
**No answer**	**Basic monitoring (NIBP, ECG)**	**Basic monitoring (Invasive blood pressure, ECG)**	**Advanced non-invasive hemodynamic monitoring**	**Advanced invasive monitoring**	**Echocardiography**			
35 (23%)	19 (12%)	80 (52%)	2 (1%)	9 (6%)	9 (6%)			
**Which are the indications for advanced hemodynamic monitoring hemorrhagic stroke patients (i.e., the use of more than invasive arterial blood pressure)?**								
**No answer**	**In all patients admitted to the ICU**	**In patients requiring drugs to increase or reduce arterial blood pressure**	**In patients with cardiovascular (i.e., pulmonary edema, cardiogenic shock, etc.) (neuroworsening)**	**In patients with neurological complications (i.e., neuroworsening)**	**Never**	**Other**		
35 (23%)	15 (10%)	40 (26%)	54 (35%)	2 (1%)	5 (3%)	3 (2%)		
**How do you manage hypotension in patients with hemorrhagic stroke?**								
**No answer**	**Mean arterial pressure-based protocol**	**No specific protocol**	**Cardiac output-based protocol**	**Fluids**				
35 (23%)	82 (53%)	25 (16%)	7 (5%)	5 (3%)				
**Which drugs are used as first line to increase blood pressure in your ICU?**								
**No answer**	**Norepinephrine**	**Vasopressin**	**Dopamine**	**Epinephrine**	**Metaraminol**	**Dobutamine**		
35 (23%)	114 (74%)	2 (1%)	1 (< 1%)	1 (< 1%)	1 (< 1%)	1 (< 1%)		
**Which drugs are used as second line to increase blood pressure in your ICU?**								
**No answer**	**Vasopressin**	**Epinephrine**	**Dobutamine**	**Norepinephrine**	**Dopamine**	**Phenylephrine**		
35 (23%)	61 (38%)	24 (16%)	22 (14%)	8 (5%)	4 (3%)	1 (< 1%)		
**Which drugs are used as first line to decrease blood pressure in your ICU?**								
**No answer**	**Beta blockers**	**Calcium channel blockers**	**Clonidine**	**Urapidil**	**Nitrates**	**ACE inhibitors**	**Angiotensin receptor antagonists**	
35 (23%)	33 (21%)	23 (15%)	16 (10%)	12 (8%)	7 (5%)	1 (< 1%)	1 (< 1%)	
**Which drugs are used as second line to decrease blood pressure in your ICU?**								
**No answer**	**Beta blockers**	**Clonidine**	**Calcium channel blockers**	**ACE inhibitors**	**Urapidil**	**Nitrates**	**Angiotensin receptor antagonists**	
35 (23%)	35 (23%)	32 (21%)	22 (14%)	10 (6%)	12 (8%)	7 (5%)	1 (< 1%)	
**How frequently do you monitor fluid balance?**								
**No answer**	**Every hour**	**Every 2 h**	**Every 4 h**	**Every 12 h**	**Every 24 h**			
35 (23%)	48 (31%)	18 (12%)	9 (6%)	23 (15%)	21 (14%)			
**Which is your net daily fluid balance target?**								
**No answer**	**Negative**	**Neutral**	**Positive**	**Other**				
35 (23%)	9 (6%)	91 (59%)	16 (10%)	3 (2%)				
**Which is the first-line fluid therapy you usually use?**								
**No answer**	**Ringer lactate**	**Normal saline**	**Hypertonic saline**	**Plasmalyte**	**Other**			
35 (23%)	52 (33%)	43 (28%)	7 (5%)	6 (4%)	11 (7%)			

ICU, intensive care unit; ABP, arterial blood pressure; NIBP, non-invasive blood pressure; ECG, electrocardiogram; ACE, angiotensin-converting enzyme; CRT, capillary refill time.

### Hemodynamic management in SAH patients

According to the responders, 44% use a standardized protocol for the blood pressure management of SAH patients.

Echocardiography during the first 24 h to rule out left ventricular dysfunction is performed by 38% of the survey responders. Cardiac output monitoring is usually used in a minority of SAH patients, and CRT is usually performed in these patients by the 36% of the responders. The commonest indication for advanced monitoring in SAH patients is the development of cardiovascular complications (47%), followed by the need for blood pressure drugs (28%), neurological complications (22%) and vasospasm (20%).

The most frequently reported goals for blood pressure before aneurysm treatment are < 140/90 mmHg (31%) and < 160/90 mmHg (22%). After aneurysm treatment, in patients without vasospasm the most common blood pressure targets are MAP 90 mmHg (24%) and MAP 80 mmHg (21%). Blood pressure in patients with vasospasm is mostly managed by targeting MAP on neurological status (32%) and targeting to a MAP of 100 mmHg (15%). Hemodynamic SAH monitoring is represented by IBP and ECG in the 44% of the answers, and NIBP and ECG in the 12%. Advanced hemodynamic monitoring is rarely used (9% of the answers).

Initial management of low blood pressure is usually performed by MAP-based protocol (49%), and the preferred first-line agent in the treatment of hypotension is norepinephrine (69%).

**Fig. 2:**
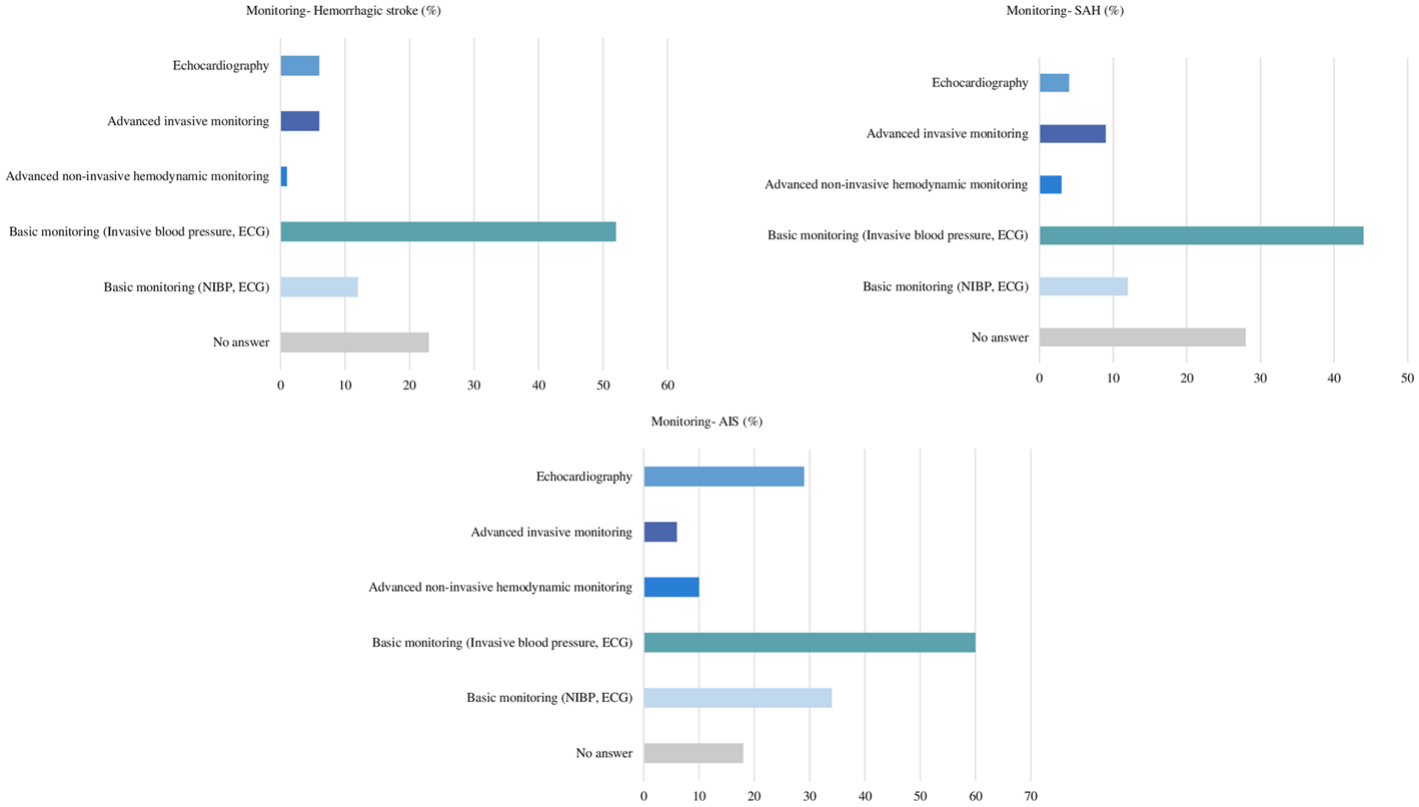
Echocardiography, continuous hemodynamic monitoring and capillary refill time use in patients with subarachnoid hemorrhage (SAH) and acute ischemic injury (AIS). Data are expressed as percentage of the overall answers obtained by the survey

Fluid balance is mostly monitored every hour (29%) and a neutral daily fluid balance is targeted by most participants (44%), a positive balance by 38 answerers (25%), and a negative balance by five (3%) survey responders. Fluids most commonly used in these patients include normal saline (30%) and Ringer lactate (28%) (Table 4 and Fig. 1) (Fig. 2).

**Table 4:** Questions and answers on subarachnoid hemorrhage

Which is the percentage of patients who undergo aneurysm clipping/year in your institution?							
**No answer**	** < 10%**	**10–30%**	**30–50%**	**50%**			
43 (28%)	35 (23%)	29 (19%)	36 (23%)	11 (7%)			
**Which is the percentage of SAH that undergo aneurism coiling/year in your institution?**							
**No answer**	** < 10%**	**10–30%**	**30–50%**	**50%**			
43 (28%)	21 (14%)	15 (10%)	27 (18%)	48 (31%)			
**Do you have a standardized protocol for arterial blood pressure management in patients with subarachnoid hemorrhage?**							
**No answer**	**Yes**	**No**					
43 (28%)	67 (44%)	44 (29%)					
**Do you routinely perform an echocardiography at the bedside in SAH patients during the first 24 h of admission to rule out possible acute LV dysfunction?**							
**No answer**	**Yes**	**No**	**Selected cases**				
43 (28%)	59 (38%)	42 (27%)	10 (6%)				
**How many SAH patients receive standard cardiac output monitoring in your institution?**							
**No answer**	** < 20%**	**30–50%**	**50%**				
43 (28%)	67 (44%)	26 (17%)	18 (12%)				
**Do you usually assess CRT at the bedside in SAH patients?**							
**No answer**	**Yes**	**No**					
43 (28%)	55 (36%)	56 (36%)					
**Which is the arterial blood pressure target that you use in patients before aneurism treatment?**							
**No answer**	** < 140/90**	** < 160/90**	**185/110**	**Reduction of 15% of the initial arterial blood pressure**	**MAP-based protocols**	**Other**	
43 (28%)	47 (31%)	34 (22%)	4 (3%)	9 (6%)	11 (7%)	6 (4%)	
**Which is the arterial blood pressure target that you use in patients after aneurism treatment without vasospasm?**							
**No answer**	**MAP + 10 mmHg compared to baseline**	**MAP 80 mmHg**	**MAP 90 mmHg**	**MAP 100 mmHg**	**Other**		
43 (28%)	15 (10%)	33 (21%)	37 (24%)	8 (5%)	18 (12%)		
**Which is the arterial blood pressure target that you use in patients in patients with vasospasm?**							
**No answer**	**MAP + 10 mmHg compared to baseline**	**MAP 80 mmHg**	**MAP 90 mmHg**	**MAP 100 mmHg**	**MAP target on neurological status**	**Other**	
43 (28%)	13 (8%)	7 (5%)	10 (6%)	23 (15%)	50 (32%)	8 (5%)	
**Which hemodynamic monitoring do you use in case of patient with subarachnoid hemorrhage with no signs of vasospasm?**							
**No answer**	**Basic monitoring (NIBP, ECG)**	**Basic monitoring (Invasive blood pressure, ECG)**	**Advanced non-invasive hemodynamic monitoring**	**Advanced invasive monitoring**	**Echocardiography**		
43 (28%)	18 (12%)	68 (44%)	5 (3%)	14 (9%)	6 (4%)		
**Which are the indications for advanced hemodynamic monitoring hemorrhagic stroke patients (i.e., the use of more than invasive arterial blood pressure)?**							
**No answer**	**In all patients admitted to the ICU**	**In patients requiring drugs to increase or reduce arterial blood pressure**	**In patients with cardiovascular (i.e., pulmonary edema, cardiogenic shock, etc.) (neuroworsening)**	**In patients with neurological complications (i.e., neuroworsening)**	**Vasospasm**	**Other**	**Never**
43 (28%)	12 (8%)	43 (28%)	73 (47%)	34 (22%)	31 (20%)	2 (1%)	3 (2%)
**How do you manage hypotension in SAH patients?**							
**No answer**	**Mean arterial pressure-based protocol**	**No specific protocol**	**Cardiac output-based protocol**	**Fluids**			
43 (28%)	76 (49%)	22 (14%)	7 (5%)	6 (4%)			
**Which drugs are used as first line to increase blood pressure in your ICU?**							
**No answer**	**Norepinephrine**	**Vasopressin**	**Dobutamine**	**Epinephrine**	**Metaraminol**		
43 (28%)	107 (69%)	3 (2%)	1 (< 1%)	1 (< 1%)	1 (< 1%)		
**Which drugs are used as second line to increase blood pressure in your ICU?**							
**No answer**	**Vasopressin**	**Epinephrine**	**Dobutamine**	**Norepinephrine**	**Dopamine**		
43 (28%)	51 (33%)	21 (14%)	21 (14%)	11 (7%)	7 (5%)		
**How frequently do you monitor fluid balance?**							
**No answer**	**Every hour**	**Every 2 h**	**Every 4 h**	**Every 12 h**	**Every 24 h**		
43 (28%)	45 (29%)	21 (14%)	7 (5%)	20 (13%)	18 (12%)		
**Which is your net daily fluid balance target?**							
**No answer**	**Negative**	**Neutral**	**Positive**				
43 (28%)	5 (3%)	68 (44%)	38 (25%)				
**Which is the first-line fluid therapy you usually use?**							
**No answer**	**Normal saline**	**Ringer lactate**	**Hypertonic saline**	**Plasmalyte**	**Albumin**	**Other**	
43 (28%)	46 (30%)	43 (28%)	6 (4%)	5 (3%)	1 (< 1%)	10 (6%)	

ICU, intensive care unit; SAH, subarachnoid hemorrhage; CRT, capillary refill time; ABP, arterial blood pressure; NIBP, non-invasive blood pressure; ECG, electrocardiogram; ACE, angiotensin-converting enzyme.

## Discussion

Given the importance of hemodynamic monitoring and management in neurocritical care patients, we conducted this cross-sectional study to describe the current practice in hemodynamic management of patients with acute brain injury admitted to ICU. The results of the study may be summarized as follows: (1) the answers were mostly obtained from European centers with medium-to-high surge capacity, managing a high number of the considered neurological diseases; (2) at least one-third of the participants use echocardiography to investigate cardiac function of the patients during the first 24 h from admission, whereas advanced hemodynamic monitoring is rarely routinely adopted, being dedicated to those patients with neurological complications or cardiovascular impairment. CRT is used by about 40% of the participants; (3) only half of the participants use a standardized protocol for blood pressure management in AIS, ICH and SAH patients; (4) MAP or SAP-based protocols are adopted to titrate systemic pressure targets; however, these targets are not universally accepted and consistent across centers; (5) norepinephrine is widely the most commonly adopted drug as first-line drug for managing hypotensive events.

Although hemodynamic management in cerebrovascular diseases is known to be crucial to minimize secondary brain damage, hemodynamic goals that should be achieved are still debated [17]. The clinical pathways to reach these clinical targets (i.e., drugs, fluids) and the hemodynamic monitoring systems that should be used to track them are not well defined. The role of the echocardiography in ICU has changed in the last decades, becoming patient-oriented, performed and interpreted by the intensivist to customize the therapy at the bedside by reassessing the effects of the strategies adopted [18]. The responses to this survey confirm the increasing use of this technique also in neurocritically ill patients.

In brain injured patients, systemic blood pressure is the most defined hemodynamic target, and an invasive monitoring is applied in selected cases of AIS, ICH and SAH (Tables 2, 3, 4). Both in AIS and ICH, blood pressure thresholds may vary accordingly to center protocols on initial management, to the time course of the disease and to the interventions performed (mechanical thrombectomy vs systemic thrombolysis). Although blood pressure threshold is the most studied hemodynamic target evaluated in literature, the survey participants declare a wide range of target MAP thresholds adopted (the most consistent answer was < 160/90 mmHg both before and after mechanical thrombectomy). Also, preferred first-line agents to lower blood pressure are different (more frequently urapidil and beta-blockers). By contrast, low blood pressure management is better standardized, with most participants using a MAP-driven protocol using norepinephrine.

In AIS and ICH patients, standard hemodynamic management is the most oriented towards a strict control of blood pressure. In SAH patients, the findings of the survey are more heterogeneous. Besides invasive BP, echocardiography, CRT and advanced invasive hemodynamic monitoring are frequently used in SAH both in the initial phase of the disease and the vasospasm phase. One possible explanation is that in the early phase, echocardiography and advanced invasive monitoring are used to stabilize patients with cardiac stunning, frequently occurring in SAH patients. In the vasospasm phase, advanced monitoring and sequential echoes are frequently used, with the aim of increase MAP (and obtain MAP variations thresholds over time in SAH), while avoiding systemic complications. Moreover, the participants showed a more liberal attitude towards the fluid balance management in the vasospasm phase.

Overall, fluid therapy approach may be considered conservative in these patients, with most centers aiming at a neutral fluid balance. This finding may be explained by the intention of avoiding further swelling of the cerebral area surrounding lesion. A recent large prospective, multicentre, trial enrolling more than 2000 patients in two cohorts of traumatic brain injured patients, showed that a mean positive daily fluid balance was associated with higher ICU mortality per 0.1 L increase [19]. In SAH patients, few trials investigated the clinical effect of different hemodynamic strategies aimed at reducing cerebral vasospasm incidence, and a recent metanalysis showed low-quality evidence to support the use of advanced hemodynamic monitoring in selected SAH patients [20–22]. Regarding the type of fluid used, only for SAH patients the saline has been considered the first option (as compared to Ringer lactate for ICH and AIS patients). A recent large randomized-controlled trial showed a potential benefit of balanced solutions use for neurological patients, confirming the importance of the type of fluid adopted for fluid resuscitation/maintenance, irrespective of the hemodynamic targets [23].

Advanced invasive and non-invasive hemodynamic monitoring using echocardiography is not frequently used; advanced monitoring is reserved to AIS and ICH patients developing cardiovascular or neurological complications, but not routinely used as standard management. This tool is widely adopted to obtain an initial assessment, but not as a part of the sequential dynamic monitoring system. However, the role of cardiac output in influencing cerebral blood flow is not widely acknowledged, especially regarding systemic blood pressure management [24]. As confirmation, a recent systematic review and meta-analysis including SAH patients identified some low-quality studies supporting advanced hemodynamic monitoring to guide clinical management [22].

### Limitations and future directions

This survey presents several limitations which must be mentioned. The external validation of this survey is intrinsically limited by the bias regarding the selection of respondents and the accuracy of the questions in defining a specific issue. For this reason, it may hardly be considered as a complete snapshot of daily clinical practice, since most of the answers are obtained from very skilled centers, overrepresenting for a particular subgroup of physicians.

Respondents from specialized neuro ICUs were included together with those working in general ICUs, yielding more generalizable findings. This survey refers only to physicians’ clinical practice in hemodynamic management of acute brain injuries without including patients’ data and only considering ICU management. Finally, the response rate cannot be calculated considering the design of the study, since participants were encouraged to involve more respondents locally.

**Fig. 3:**
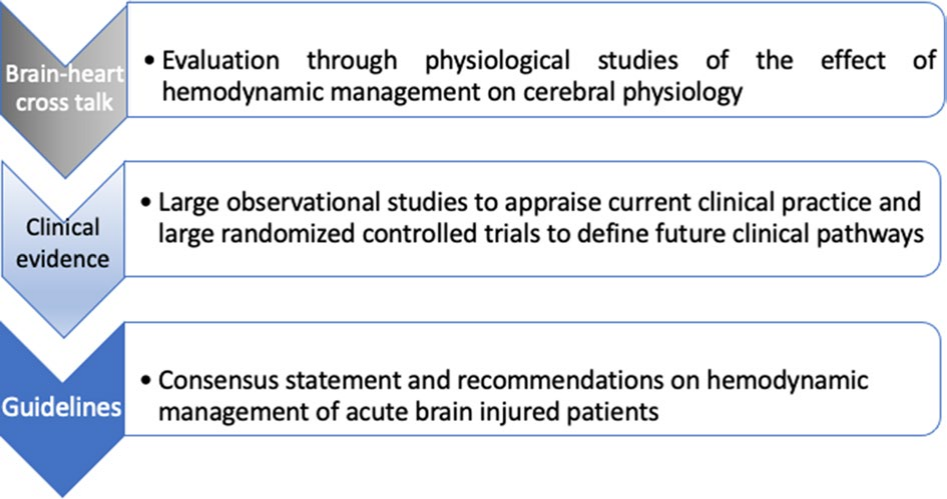
Main steps for a research agenda for the hemodynamic management of acute brain injured patients

Highlighting the heterogeneity of hemodynamic monitoring and the lack of specific protocols of treatment on fluid management in neurocritical care patients, this survey may be also considered as a starting point to guide a research agenda in this field (Fig. 3). Starting from the evidence retrieved by several observational studies, large and specific randomized-controlled trials aiming at evaluating the clinical effect regarding the use of advanced hemodynamic monitoring and specific fluid/pressure targets should be designed in this field.

## Conclusions

This ESICM survey shows important heterogeneity in the approach of hemodynamic monitoring and management of brain injured patients, including pharmacologic and non-pharmacological strategies. This survey may pave the way for the development of a research agenda, in the field of hemodynamic management of this population of patients, where evidence is importantly lacking.

## Data Availability

The datasets used and/or analyzed during the current study are available from the corresponding author on reasonable request.

## References

[1] Trivedi M, Coles JP (2009). Blood pressure management in acute head injury. J Intensive Care Med.

[2] Lee K, Choi HA, Edwards N, Chang T, Sladen RN (2014). Perioperative critical care management for patients with aneurysmal subarachnoid hemorrhage. Korean J Anesthesiol.

[3] Diringer MN, Axelrod Y (2007). Hemodynamic manipulation in the neuro-intensive care unit: Cerebral perfusion pressure therapy in head injury and hemodynamic augmentation for cerebral vasospasm. Curr Opin Crit Care.

[4] van der Jagt M (2016). Fluid management of the neurological patient: A concise review. Crit Care.

[5] Connolly ES, Rabinstein AA, Carhuapoma JR, Derdeyn CP, Dion J, Higashida RT (2012). Guidelines for the management of aneurysmal subarachnoid hemorrhage: A guideline for healthcare professionals from the American heart association/American stroke association. Stroke.

[6] Diringer MN, Bleck TP, Claude Hemphill J (2011). Critical care management of patients following aneurysmal subarachnoid hemorrhage: Recommendations from the neurocritical care society's multidisciplinary consensus conference. Neurocrit Care.

[7] Jauch EC, Saver JL, Adams HP, Bruno A, Connors JJ, Demaerschalk BM (2013). Guidelines for the early management of patients with acute ischemic stroke: A guideline for healthcare professionals from the American heart association/American stroke association. Stroke.

[8] Le Roux P, Menon DK, Citerio G, Vespa P, Bader MK, Brophy GM (2014). Consensus summary statement of the international multidisciplinary consensus conference on multimodality monitoring in neurocritical care : A statement for healthcare professionals from the neurocritical care society and the European society of intensive care medicine. Intensive Care Med.

[9] Taccone FS, Citerio G. Participants in the International Multi-disciplinary Consensus Conference on Multimodality M: Advanced monitoring of systemic hemodynamics in critically ill patients with acute brain injury. Neurocrit Care 2014;21:S38–63. 10.1007/s12028-014-0033-5 25208672

[10] Anderson CS, Huang Y, Lindley RI, Chen X, Arima H, Chen G (2019). Intensive blood pressure reduction with intravenous thrombolysis therapy for acute ischaemic stroke (enchanted): An international, randomised, open-label, blinded-endpoint, phase 3 trial. Lancet.

[11] Qureshi AI, Foster LD, Lobanova I, Huang W, Suarez JI (2020). Intensive blood pressure lowering in patients with moderate to severe grade acute cerebral hemorrhage: Post hoc analysis of antihypertensive treatment of acute cerebral hemorrhage (atach)-2 trial. Cerebrovasc Dis.

[12] Qureshi AI, Palesch YY, Barsan WG, Hanley DF, Hsu CY, Martin RL (2016). Intensive blood-pressure lowering in patients with acute cerebral hemorrhage. N Engl J Med.

[13] Greenberg SM, Ziai WC, Cordonnier C, Dowlatshahi D, Francis B, Goldstein JN (2022). guideline for the management of patients with spontaneous intracerebral hemorrhage: A guideline from the American heart association/American stroke association. Stroke.

[14] Lee KH, Lukovits T, Friedman JA (2006). "Triple-h" therapy for cerebral vasospasm following subarachnoid hemorrhage. Neurocrit Care.

[15] Mutoh T, Kazumata K, Ishikawa T, Terasaka S (2009). Performance of bedside transpulmonary thermodilution monitoring for goal-directed hemodynamic management after subarachnoid hemorrhage. Stroke.

[16] Muench E, Horn P, Bauhuf C, Roth H, Philipps M, Hermann P (2007). Effects of hypervolemia and hypertension on regional cerebral blood flow, intracranial pressure, and brain tissue oxygenation after subarachnoid hemorrhage. Crit Care Med.

[17] Messina A, Villa F, Cecconi M (2022). Challenges in the hemodynamic management of acute nontraumatic neurological injuries. Curr Opin Crit Care.

[18] Vignon P, Begot E, Mari A, Silva S, Chimot L, Delour P (2018). Hemodynamic assessment of patients with septic shock using transpulmonary thermodilution and critical care echocardiography: A comparative study. Chest.

[19] Wiegers EJA, Lingsma HF, Huijben JA, Cooper DJ, Citerio G, Frisvold S (2021). Fluid balance and outcome in critically ill patients with traumatic brain injury (center-tbi and ozenter-tbi): A prospective, multicentre, comparative effectiveness study. Lancet Neurol.

[20] Rondeau N, Cinotti R, Rozec B, Roquilly A, Floch H, Groleau N (2012). Dobutamine-induced high cardiac index did not prevent vasospasm in subarachnoid hemorrhage patients: A randomized controlled pilot study. Neurocrit Care.

[21] Rinkel GJ, Feigin VL, Algra A, van Gijn J (2004). Circulatory volume expansion therapy for aneurysmal subarachnoid haemorrhage. Cochrane Database Syst Rev.

[22] Simonassi F, Ball L, Badenes R, Millone M, Citerio G, Zona G (2021). Hemodynamic monitoring in patients with subarachnoid hemorrhage: A systematic review and meta-analysis. J Neurosurg Anesthesiol.

[23] Semler MW, Self WH, Rice TW (2018). Balanced crystalloids versus saline in critically ill adults. N Engl J Med.

[24] Drummond JC (2020). Cardiac output: The neglected stepchild of the cerebral blood flow physiology family. J Neurosurg Anesthesiol.

